# Development and Characterization of a Novel Live Attenuated Vaccine Against Enteric Septicemia of Catfish

**DOI:** 10.3389/fmicb.2018.01819

**Published:** 2018-08-07

**Authors:** Hossam Abdelhamed, Mark L. Lawrence, Attila Karsi

**Affiliations:** Department of Basic Sciences, College of Veterinary Medicine, Mississippi State University, Starkville, MS, United States

**Keywords:** *Edwardsiella ictaluri*, type six secretion sysytem, T6SS, *evpB*, live attenuated vaccine

## Abstract

*Edwardsiella ictaluri* is a Gram-negative intracellular pathogen causing enteric septicemia of channel catfish (ESC). Type six secretion system (T6SS) is a sophisticated nanomachine that delivers effector proteins into eukaryotic host cells as well as other bacteria. In the current work, we in-frame deleted the *E. ictaluri* *evpB* gene located in the T6SS operon by allelic exchange. The safety and efficacy of *Ei*Δ*evpB* as well as Aquavac-ESC, a commercial vaccine manufactured by Intervet/Merck Animal Health, were evaluated in channel catfish (*Ictalurus punctatus*) fingerlings and fry by immersion exposure. Our results showed that the *Ei*Δ*evpB* strain was avirulent and fully protective in catfish fingerlings. The *Ei*Δ*evpB* strain was also safe in catfish fry, and immersion vaccination with *Ei*Δ*evpB* at doses 10^6^ and 10^7^ CFU/ml in water resulted in 34.24 and 80.34% survival after wild-type immersion challenge compared to sham-vaccinated fry (1.79% survival). Catfish fry vaccinated with *Ei*Δ*evpB* at doses 10^6^, 10^7^, and 10^8^ CFU/ml in water exhibited dose-dependent protection. When compared with Aquavac-ESC, *Ei*Δ*evpB* provided significantly higher protection in catfish fingerlings and fry (*p* < 0.05). Results indicate that the *Ei*Δ*evpB* strain is safe and can be used to protect catfish fingerlings and fry against *E. ictaluri*.

## Introduction

Channel catfish (*Ictalurus punctatus*) is the most significant aquaculture commodity in the United States, and *Edwardsiella ictaluri* causes enteric septicemia of channel catfish (ESC) (Hawke, 1979). The disease occurs as acute enteric septicemia or chronic encephalitis (Shotts et al., 1986). Use of antibiotic-added feed (Smith et al., 1994; Plumb et al., 1995) and feed restriction (Wise et al., 2004) are traditional means used to control ESC. Although these practices could reduce mortalities, feed restriction results in reduced production through lost feeding days. Medicated feed is expensive, useful only in fish that accept feed, and could yield antibiotic-resistant strains.

Vaccination is a vital prophylactic strategy for prevention of bacterial diseases in aquaculture (Shoemaker et al., 2009; Villumsen et al., 2014). Because *E. ictaluri* species has been shown to be very homogeneous (Plumb and Vinitnantharat, 1989; Bertolini et al., 1990), a vaccine strain could potentially provide wide-range of protection against different *E. ictaluri* strains in different fish species. The commercial ESC vaccine Aquavac-ESC (RE-33) was developed by serial passage in increasing concentrations of rifampicin (Klesius and Shoemaker, 1997). Aquavac-ESC is safe in catfish fry (Klesius and Shoemaker, 1999), but it has not been accepted widely due to marginal economic returns (Bebak and Wagner, 2012). Another live attenuated *E. ictaluri* vaccine was developed by serial passage on media containing increasing concentrations of rifamycin, which protected catfish fingerlings when added in feed and administered orally (Wise et al., 2015). Under current catfish production practices, catfish fry are transferred from hatchery to nursery ponds when they are 1–2-week-old. Therefore, there is an urgent need for an effective ESC vaccine that can be delivered to catfish fry before their release into nursery ponds.

Type six secretion system (T6SS) is a virulence factor for many pathogenic bacteria (Rao et al., 2004; Pukatzki et al., 2006). This system is highly conserved and widely distributed in Gram-negative bacteria as one or more copies (Bingle et al., 2008). T6SS delivers protein effectors into the periplasm of the target cells directly upon cell-to-cell contact. Therefore, contributing to different processes ranging from inter-bacterial killing to pathogenesis. The number of genes encoded within T6SS clusters usually varies between 16 and 38 genes (Cascales, 2008; Murdoch et al., 2011), with a minimal set of 13 genes required to assemble a functional T6SS (Boyer et al., 2009; Lin et al., 2013). T6SS is also required to kill other bacterial cells by secreting anti-bacterial proteins (Ho et al., 2014).

Our previous proteomics study showed that EvpB protein is differentially regulated during *in vitro* iron-restricted conditions (Dumpala et al., 2015). Thus, we hypothesized that EvpB protein could have a crucial role in the T6SS of *E. ictaluri*. In the current work, we report the construction of an *evpB* in-frame deletion mutant and its vaccine potential in catfish fingerlings and fry.

## Materials and Methods

### Bacterial Strains, Plasmids, and Growth Conditions

Bacterial strains and plasmids used in this work are listed in **Table 1**. Wild-type *E. ictaluri* strain 93–146 (*Ei*WT) was cultured in brain heart infusion (BHI) agar or broth (Difco, Sparks, MD, United States) and incubated at 30°C throughout the study. *E. coli* strains CC118λ*pir* and SM10λ*pir* were cultured on Luria-Bertani (LB) agar or broth (Difco, Sparks, MD, United States) and incubated at 37°C throughout the study. When required, media were supplemented with the following antibiotics and reagents: ampicillin (amp: 100 mg/ml), colistin sulfate (col: 12.5 mg/ml), sucrose (5%), and mannitol (0.35%) (Sigma-Aldrich, St. Louis, MO, United States).

**Table 1:** Bacterial strains and plasmids.

Bacterial strain	Strains	References
*Edwardsiella ictaluri*		
*93–146*	Wild type; pEI1^+^; pEI2^+^; Col^r^	Lawrence et al., 1997
*Ei*Δ*evpB*	*93–146 derivative;* pEI1^+^; pEI2^+^; Col^r^; Δ*evpB*	This study
*Ei*Δ*evpB+*p*EievpB*	*Ei*Δ*evpB*, p*EievpB*	This study
*Escherichia coli*		
CC118aaa*pir*	Δ*(ara-leu); araD;*Δ*lacX74; galE; galK; phoA20; thi-1; rpsE; rpoB; argE(Am); recAl; aaapirR6K*	Herrero et al., 1990
SM10aaa*pir*	*thi; thr; leu; tonA; lacY; supE; recA;::RP4-2-Tc::Mu; Kmr; aaapirR6K*	Miller and Mekalanos, 1988
Plasmids		
pMEG-375	8142 bp, Amp^r^, Cm^r^, *lacZ*, R6K *ori*, *mob incP*, *sacR* *sacB*	Dozois et al., 2003
p*Ei*Δ*evpB*	10507 bp, pMEG-375,::Δ*evpB*	This study
pBBR1-MCS4	4950 bp, broad-host-range expression vector; Ap^r^	Kovach et al., 1995
p*EievpB*	pBBR1-MCS4 carrying *evpB*	This study


### Sequence Analysis

The nucleotide sequences of the T6SS operon were obtained from the *E. ictaluri* strain 93–146 genome (GenBank accession: CP001600) (Williams et al., 2012). The Basic Local Alignment Search Tool (BLAST) was used to determine the sequence of the *evpB* open reading frame and adjacent sequences.

### Construction of *Ei*Δ*evpB* In-frame Deletion Mutant

In-frame deletion of the *E. ictaluri* *evpB* gene (NT01EI_RS11895) was accomplished by following the procedures described previously (Abdelhamed et al., 2013). Briefly, 1,210 bp upstream and 1,155 bp downstream regions of *evpB* were amplified from *E. ictaluri* strain 93–146 genomic DNA with A/B and C/D primer pairs (**Table 2**), respectively. These PCR products were mixed, diluted, and used as template in a splicing overlap extension PCR (Horton et al., 1990) with the A/D primers to generate Δ*evpB* deletion fragment (2,365 bp). The resulting Δ*evpB* deletion fragment was cloned into pMEG-375 suicide plasmid. The resulting plasmid, p*Ei*Δ*evpB*, was transformed into *E. ictaluri* by conjugation, and transformants were selected on BHI agar containing Amp and Col at 30°C for 2 days. To allow the second homologous recombination, a single Amp-resistant merodiploid colony was plated on BHI agar with sucrose and mannitol and incubated at 30°C for 3 days. Amp-sensitive colonies were screened by colony PCR using the A/D primers, and further confirmation was done by sequencing of A/D fragment.

**Table 2:** Primers used to generate and verify in-frame deletion of the *E. ictaluri* *evpB* gene.

Primers		Sequence^a^	RE^b^
*EievpB*-F01	A	AA**TCTAGA**GGACGACTCACCTCCGTTATC	*Xba*I
*EievpB*-R189	B	TACGTCACCGGAAACTGTCAC	
*EievpB*-F1375	C	GTGACAGTTTCCGGTGACGTAGATGTCAGCGATATTCCAGGT	
*EievpB*-R01	D	AA**TCTAGA**GTTGATCGCTGTACCGATGTC	*Xba*I
*EievpB*-Seq		GCTTCCCAAGCTGAAAGAAC	
*EievpB*-F01-Comp		AA**CCCGGG**ATGAGCGAACAGAACTTGC	*Sma*I
*EievpB*-R01-Comp		AA**TCTAGA**ATCGGCGACCAAACGTAAAG	*Xba*I


*^*a*^Bold letters at the 5^′^ end of the primer sequence represent restriction enzyme (RE) site added. AA nucleotides were added to the end of primers containing a RE site to increase the efficiency of enzyme cut. Underlined bases in primer C indicate reverse complemented primer B sequence.^*b*^RE stands for restriction enzyme.*

### Complementation of the *evpB* Gene

The 1,464 bp open reading frame of the *evpB* gene was amplified using primers listed in **Table 2**. The amplicon was cloned into a pBBR1-MCS4 plasmid (Kovach et al., 1995) at the *Sma*I and *Xba*I restriction sites. The resulting plasmid, p*EievpB*, was transferred to *Ei*Δ*evpB* by conjugation. Successful transformation was verified by observing plasmid profile of *Ei*Δ*evpB*. The resulting strain was designated as *Ei*Δ*evpB+*p*EievpB*.

### Determination of Safety and Efficacy of *Ei*Δ*evpB* in Catfish Fingerlings

All fish experiments were approved by the Institutional Animal Care and Use Committee at Mississippi State University (protocol numbers: 12–042, 15–043, and 17–288). Virulence and vaccine efficacy of the *Ei*Δ*evpB* strain were assessed, as described (Abdelhamed et al., 2013). Briefly, 240 specific-pathogen-free (SPF) channel catfish fingerlings (13.88 ± 0.27 cm, 27.77 ± 1.04 g) were stocked in 40 l flow-through tanks (20 fish/tank) with constant aeration and allowed to acclimate for 1 week. Water temperature was maintained at 26 ± 2°C during the experiment. The tanks were randomly assigned into three groups, and each group contained four replicates. The three groups were *Ei*Δ*evpB,* *Ei*WT (positive control), and BHI (sham control). The fish were vaccinated by immersion for 1 h in water containing approximately 3.32 × 10^7^ CFU/ml, and then flow-through conditions were resumed. Mortalities were recorded daily, and the presence of *E. ictaluri* was confirmed by streaking anterior kidney onto BHI plates. At 21-days post-immunization, the vaccinated fish were challenged with *Ei*WT (3.83 × 10^7^ CFU/ml in water) by immersion for 1 h as described above. Mortalities were recorded daily.

### Determination of Safety and Efficacy of *Ei*Δ*evpB* in Catfish Fry

Nine hundred 14-day-old SPF channel catfish fry were stocked in 18 tanks (50 fry/tank). Tanks were randomly assigned to six treatment groups with three replicates per group. Treatment groups consisted of high (3.32 × 10^7^ CFU/ml in water) and low (3.32 × 10^6^ CFU/ml in water) doses of *Ei*Δ*evpB,* *Ei*WT, and BHI. Immersion vaccination was conducted same as fingerling challenge described above. At 21 days post-vaccination, fry were challenged with *Ei*WT by immersion exposure at approximately 3.10 × 10^7^ CFU/ml in water. Mortalities were recorded daily.

### Evaluation of Various Challenge Doses of *Ei*Δ*evpB* in Catfish Fry

Vaccine efficacies of three separate doses of *Ei*Δ*evpB* were evaluated in 7-day-old fry to determine the optimal dose. Briefly, 750 fry were stocked into 15 tanks (50 fry/tank). The tanks were divided into five groups with three replicates per group. Vaccinated groups consisted of three doses of *Ei*Δ*evpB* (3.72 × l0^6^, 3.72 × l0^7^, and 3.72 × l0^8^ CFU/ml in water), *Ei*WT (positive control), and BHI (sham control). Fish were monitored daily, and mortalities were recorded from each tank. After 30 days post-vaccination, fry were challenged with the *Ei*WT by immersion in water (3.80 × 10^7^ CFU/ml) for 1 h. Mortalities were recorded daily.

### Comparison of *Ei*Δ*evpB* and Aquavac-ESC in Catfish Fingerlings

Vaccine efficacy of *Ei*Δ*evpB* strain was compared with Aquavac-ESC in catfish fingerlings. Briefly, 320 channel catfish fingerlings (7.75 ± 0.08 cm, 4.50 ± 0.014 g) were stocked into 16 tanks (20 fish/tank). Each group included four replicate tanks. Vaccination groups consisted of Aquavac-ESC, *Ei*Δ*evpB*, *Ei*WT (positive control), and BHI (sham control). Fingerlings were vaccinated by immersion in water containing approximately 4.5 × 10^7^ CFU/ml for 1 h. Fish were monitored, and dead fish were removed daily. After 21 days, immunized fish were challenged with *Ei*WT by immersion in water with 3.80 × 10^7^ CFU/ml for 1 h. Mortalities were recorded daily.

### Comparison of *Ei*Δ*evpB* and Aquavac-ESC in Catfish Fry

Vaccine efficacy of *Ei*Δ*evpB* was compared with Aquavac-ESC in 14-days post-hatch fry. Briefly, 800 channel catfish fry were stocked into 16 tanks (50 fish/tank). Each group included four replicate tanks. Vaccination groups consisted of *Ei*Δ*evpB* strain, Aquavac-ESC, *Ei*WT (positive control), and BHI (sham control). Fry were vaccinated by immersion (3.72 × 10^7^ CFU/ml in water) for 1 h. Fish were monitored, and dead fish were removed daily. After 21 days, immunized fish were challenged with *Ei*WT by immersion (3.80 × 10^7^ CFU/ml in water) for 1 h. Mortalities were recorded daily.

### Comparison of *Ei*Δ*evpB* and Complemented Strain

Virulence of *Ei*Δ*evpB+*p*Ei*evpB strain was compared to *Ei*Δ*evpB* in 12-days post-hatch fry. Briefly, 360 channel catfish fry were stocked into 12 tanks (30 fish/tank). Experimental groups consisted of *Ei*Δ*evpB*, *Ei*Δ*evpB+*p*Ei*evpB, *Ei*WT (positive control), and BHI (negative). Each group included three replicate tanks. Fry were challenged by immersion (4.60 × 10^7^ CFU/ml in water) for 1 h. Mortalities were recorded daily.

### Statistical Analysis

The percent mortality and survival values were arcsine transformed, and pairwise comparison of the means was performed with Tukey procedure. Analysis of variance (ANOVA) was done using PROC GLM in SAS for Windows v9.4 (SAS Institute, Inc., Cary, NC, United States) to assess significance. An alpha level of 0.05 was used in all analyses.

## Results

### T6SS in *E. ictaluri* Genome

**FIGURE 1:**
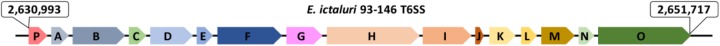
Type VI secretion system organization in the *E. ictaluri* genome. Arrows indicate the direction of transcription, and numbers at the beginning and the end indicate genomic coordinates.

Analysis of the *E. ictaluri* genome revealed the presence of a 20,724 bp operon containing 16 genes (*evpP*, plus genes from NT01EI_RS11890 to NT01EI_RS11960) that encode for the T6SS apparatus, chaperones, effectors, and regulators (**Figure 1**).

### Construction of the *Ei*Δ*evpB* Mutant

We successfully introduced an in-frame deletion to the *evpB* gene in the *E. ictaluri* chromosome. The resulting *Ei*Δ*evpB* strain contained a deletion of 1,167 bp out of 1,488 bp open reading frame (78.42%), resulting in loss of 389 amino acids from the *E. ictaluri* *evpB* gene. This in-frame deletion was verified by PCR and sequencing of the amplified fragment from the *Ei*Δ*evpB* strain.

### Virulence and Vaccine Efficacy of *Ei*Δ*evpB* in Catfish Fingerlings

**FIGURE 2:**
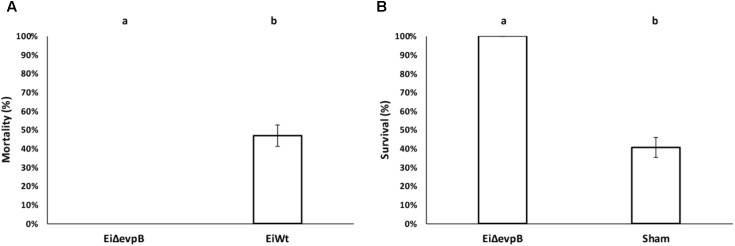
Results of virulence **(A)** and vaccine efficacy **(B)** trials of *Ei*Δ*evpB* in catfish fingerlings. Lowercase letters indicate significant differences (*p* < 0.05).

The virulence of *Ei*Δ*evpB* (0% mortality) was significantly lower than those fish challenged with *Ei*WT (46.91% mortality) (*P* < 0.5) (**Figure 2A**). At 21 days post-vaccination, the *Ei*Δ*evpB* vaccinated group had significantly higher survival compared to the sham-vaccinated group (100% vs. 40.69% survival, respectively) (*P* < 0.05) (**Figure 2B**).

### Virulence and Efficacy of *Ei*Δ*evpB* in Fry

**FIGURE 3:**
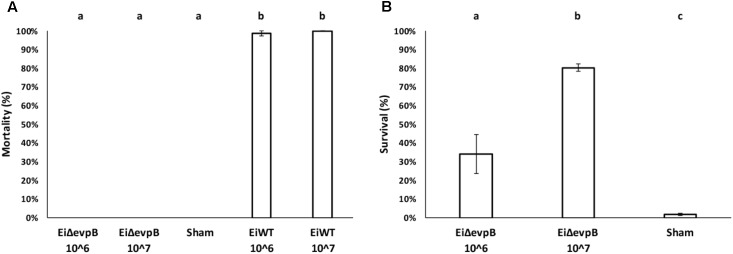
Results of virulence **(A)** and vaccine efficacy **(B)** trials in catfish fry vaccinated with two different doses of *Ei*Δ*evpB* (10^6^ and 10^7^CFU/ml in water). Lowercase letters indicate significant differences (*p* < 0.05).

No mortality was observed in the fry vaccinated with *Ei*Δ*evpB* at 10^6^ and 10^7^ CFU/ml in water. In contrast, 98.67 and 100% mortalities were observed in the fry exposed to *Ei*WT at 10^6^ and 10^7^ CFU/ml in water doses, respectively (**Figure 3A**). The fry vaccinated with *Ei*Δ*evpB* showed 34.24% survival at 10^6^ CFU/ml in water, and 80.34% survival at 10^7^ CFU/ml in water at 21 days post-vaccination. On the contrary, the sham-vaccinated group had 1.79% survival (**Figure 3B**).

### Optimal Vaccine Dose of *Ei*Δ*evpB* in Catfish Fry

**FIGURE 4:**
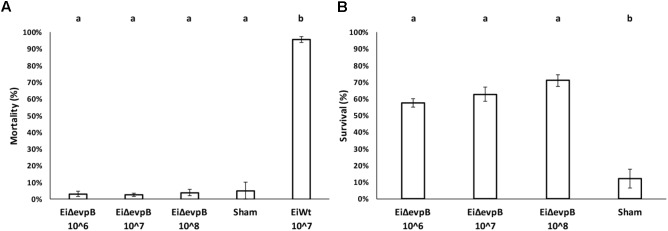
Results of virulence **(A)** and vaccine efficacy **(B)** trials of three different vaccination doses (10^6^, 10^7^, and 10^8^ CFU/ml in water) of *Ei*Δ*evpB* in catfish fry. Lowercase letters indicate significant differences (*p* < 0.05).

Mortality in 7-day old fry vaccinated with *Ei*Δ*evpB* (10^6^, 10^7^, and 10^8^ CFU/ml in water) and sham group ranged between 2.53 and 5.13%, which was not statistically different. Dead fish collected from these treatments did not show any pathology, and *E. ictaluri* was not present in the fish. On the other hand, very high mortality (95.71%) was observed in *Ei*WT exposed (10^7^ CFU/ml in water) fry (**Figure 4A**). At 30-day post-vaccination with three different doses (10^6^, 10^7^, and10^8^ CFU/ml in water), the percent survival in fry challenged with *Ei*WT were significantly higher (57.74, 62.74, and 71.06%, respectively) compared to the sham-vaccinated fry (12.16% survival) (*P* < 0.05) (**Figure 4B**).

### Comparison of *Ei*Δ*evpB* to Aquavac-ESC in Catfish Fingerling

**FIGURE 5:**
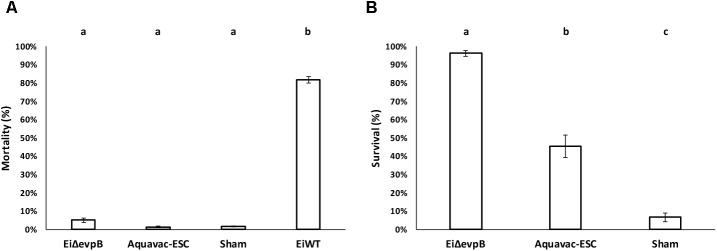
Results of virulence **(A)** and vaccine efficacy **(B)** trials of *Ei*Δ*evpB* and Aquavac-ESC in catfish fingerlings. Lowercase letters indicate significant differences (*p* < 0.05).

*Ei*Δ*evpB*, Aquavac-ESC, and sham control showed low mortalities (5.14, 1.45, and 1.67%, respectively) in catfish fingerlings. On the other hand, *Ei*WT challenge caused 81.83% mortality (**Figure 5A**). Catfish fingerlings vaccinated with *Ei*Δ*evpB* elicited significantly higher protection (96.20% survival) compared to that of Aquavac-ESC (45.51% survival) and sham-vaccinated (6.67% survival) groups (*p* < 0.05) following *Ei*WT challenge (**Figure 5B**).

### Comparison of *Ei*Δ*evpB* to Aquavac-ESC in Catfish Fry

**FIGURE 6:**
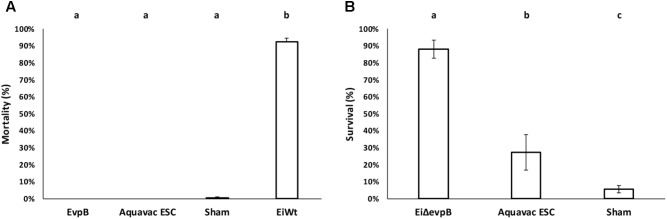
Results of virulence **(A)** and vaccine efficacy **(B)** trials of *Ei*Δ*evpB* and Aquavac-ESC in catfish fry. Lowercase letters indicate significant differences (*p* < 0.05).

*Ei*Δ*evpB* and Aquavac-ESC showed no mortalities, while negligible mortality was observed in the sham group (0.50%). *Ei*WT group exhibited very high mortality (92.21% mortality) (**Figure 6A**). These results indicated that *Ei*Δ*evpB* and Aquavac-ESC were safe in catfish fry. In the vaccine efficacy experiment, *Ei*Δ*evpB* strain elicited significantly higher protection compared to Aquavac-ESC and sham-vaccinated fish (88.10, 27.40, and 5.56% survival, respectively) (*p* < 0.05) (**Figure 6B**).

### Comparison of *Ei*Δ*evpB* to Complemented Strain

**FIGURE 7:**
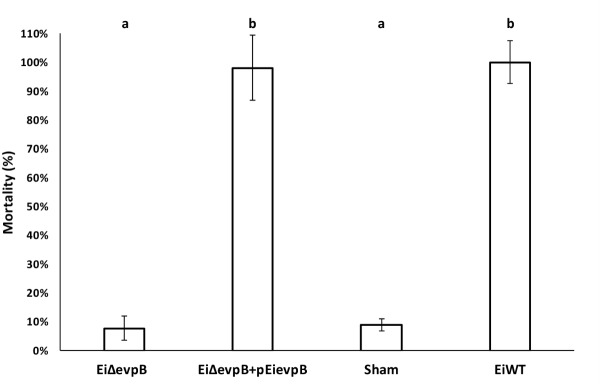
Mortalities observed in catfish fry vaccinated with *Ei*Δ*evpB*, complemented (*Ei*Δ*evpB+*p*EievpB*), sham, and *Ei*WT groups. Lowercase letters indicate significant differences (*p* < 0.05).

Complementation of *Ei*Δ*evpB* with p*Ei*evpB plasmid carrying the wild-type *evpB* gene restored the wild-type phenotype. The percent mortalities in 12-day-old fry vaccinated with *Ei*Δ*evpB*, *Ei*Δ*evpB+*p*Ei*evpB, sham, and *Ei*WT groups were 7.78, 98.02, 8.89, and 100%, respectively (**Figure 7**).

## Discussion

The primary objective of this study was to develop a live attenuated *E. ictaluri* vaccine strain based on mutation of the *evpB* gene, which is the second gene in the T6SS operon. The *E. ictaluri* EvpB protein was previously annotated as Eip55 (Moore et al., 2002), but it was not known that Eip55 was part of the T6SS. Eip55 is expressed during *E. ictaluri* infection and is antigenic to channel catfish. The percent sequence identity at the amino acid level between *E. ictaluri* and *E. tarda* EvpB (Rao et al., 2004) is high (96.5%). The first step of our work was construction of the *Ei*Δ*evpB* strain by in-frame deletion of the *evpB* gene leaving 189 bp at the 5^′^ end and 132 bp at the 3^′^ end. Mutant construction did not introduce any selective antibiotics to the *Ei*Δ*evpB* strain. Introduction of extraneous antibiotic resistance in vaccine strains is not desirable to avoid spread of antibiotic resistance genes.

The *Ei*Δ*evpB* strain was completely avirulent in catfish fingerlings. Moreover, vaccination of fingerlings with *Ei*Δ*evpB* provided full protection against subsequent challenge with *Ei*WT at 21 days post-vaccination. However, U.S. catfish production practices limit immersion vaccination to 7–14 days post-hatch. At this age, 1000s of fry are housed in hatchery tanks and can be vaccinated cost-effectively. To conform to industry practices, the *Ei*Δ*evpB* was assessed in 14-day-old fry by immersion at two different doses (10^6^ and 10^7^ CFU/ml in water). The result demonstrated that the *Ei*Δ*evpB* strain was completely attenuated in channel catfish fry at both doses. Following vaccination, we found 80.34% survival at the 10^7^ CFU/ml in water dose and 34.24% survival at the 10^6^ CFU/ml in water dose. These results demonstrate that 10^7^ CFU/ml in water dose of *Ei*Δ*evpB* could provide excellent protection levels in catfish fry against ESC.

Immunization of 7-day-old catfish fry with increasing doses of *Ei*Δ*evpB* (10^6^, 10^7^, and 10^8^ CFU/ml in water) demonstrated that the protection levels of *Ei*Δ*evpB*, although was not statistically significant, tend to be higher with increasing vaccine dose. Challenge doses as high as 10^8^ CFU/ml in water were safe under our experimental conditions, and the higher dose of *Ei*Δ*evpB* elicited better protection against *Ei*WT. However, subsequent immunizations were conducted using a dose of 10^7^ CFU/ml in water, which is a more achievable dose for commercial vaccine manufacturing.

We also compared vaccine efficacy of *Ei*Δ*evpB* to the commercial vaccine Aquavac-ESC in channel catfish fingerlings and fry by immersion. In both trials, *Ei*Δ*evpB* provided better protection than Aquavac-ESC. Besides the superior performance of *Ei*Δ*evpB* compared to Aquavac-ESC, *Ei*Δ*evpB* does not have an added antibiotic resistance gene while Aquavac-ESC is rifampicin resistant. Also, *Ei*Δ*evpB* has a known genotype, while the genetic basis for Aquavac-ESC attenuation is not described.

The results from fry and fingerling experiments showed that *evpB* is vital in *E. ictaluri* virulence, which is consistent with findings in *E. tarda* PPD130/91, where deletion of *evpB* led to reduced virulence in blue gourami and impaired replication in gourami phagocytes (Rao et al., 2004). Several T6SS proteins are important for bacterial pathogenesis. However, the function of most T6SS proteins remains unknown (Filloux et al., 2008; Silverman et al., 2012). This is the first report to our knowledge that *evpB* is required for *E. ictaluri* virulence. This is also the first study that linked T6SS and virulence in *E. ictaluri*.

The results shown here suggest that deletion of *evpB* gene provides an excellent live attenuated vaccine candidate. Live attenuated bacterial vaccines activate immune responses by mimicking the route of natural infection, possess intrinsic adjuvant properties, and can be administrated as mucosal vaccines. In the present study, only the immersion route of exposure was tested, which is the preferred vaccination method in commercial settings, because large numbers of small fish can be vaccinated quickly and cheaply (Stevenson, 1997; Chettri et al., 2013).

Live attenuated vaccines must achieve a precise balance between lack of pathogenicity and sufficient immunogenicity to provide protective immunity. An important consideration for the success of vaccination in catfish fry is that the immune system of fry may not be fully developed (Ellis, 1988). Therefore, fry may not be able to respond to vaccination effectively. The protective immunity in channel catfish is determined by cellular immune mechanisms, which generally precedes the development of humoral immunity (Shoemaker et al., 1997; Thune et al., 1997; Petrie-Hanson and Ainsworth, 2001). In previous studies, it has been reported that catfish fry failed to produce a significant antibody response before 3 weeks of age due to the poor organization of secondary lymphoid tissue (Patrie-Hanson and Jerald Ainsworth, 1999; Petrie-Hanson and Ainsworth, 2001). This could elucidate why attempts of early vaccination of fry are less likely to succeed. However, our results showed that vaccination of 2-week-old catfish fry with *Ei*Δ*evpB* could provide very high protection. It is clear that use of *Ei*Δ*evpB* at three or 4-week-old fry may provide full protection against *Ei*WT, but current catfish practices do not permit housing fry in hatcheries beyond 10–14 days.

## Conclusion

In conclusion, *Ei*Δ*evpB* described in this work is entirely safe and provides full protection for catfish fingerlings. Also, *Ei*Δ*evpB* is safe and highly protective in catfish fry. Further, *Ei*Δ*evpB* is well characterized genetically and does not carry any additional antibiotic resistance. Field trials in earthen ponds will allow us to assess the commercial potential of *Ei*Δ*evpB* under production conditions. Understanding the responses of the catfish immune system to *Ei*Δ*evpB* vaccination will help us develop a better vaccination strategy.

## Author Contributions

AK and ML supervised the study. HA, ML, and AK designed the experiments and analyzed and interpreted the data. HA and AK performed the experiments. All authors wrote and approved the manuscript.

## Conflict of Interest Statement

The authors declare that the research was conducted in the absence of any commercial or financial relationships that could be construed as a potential conflict of interest.
